# Gender Specifics of Healthy Ageing in Older Age as Seen by Women and Men (70+): A Focus Group Study

**DOI:** 10.3390/ijerph19053137

**Published:** 2022-03-07

**Authors:** Katja Schladitz, Franziska Förster, Michael Wagner, Kathrin Heser, Hans-Helmut König, André Hajek, Birgitt Wiese, Alexander Pabst, Steffi G. Riedel-Heller, Margrit Löbner

**Affiliations:** 1 Institute of Social Medicine, Occupational Health and Public Health (ISAP), Faculty of Medicine, University of Leipzig, Philipp-Rosenthal-Str. 55, 04103 Leipzig, Germany; franziska.foerster@medizin.uni-leipzig.de (F.F.); alexander.pabst@medizin.uni-leipzig.de (A.P.); steffi.riedel-heller@medizin.uni-leipzig.de (S.G.R.-H.); margrit.loebner@medizin.uni-leipzig.de (M.L.); 2 Department of Neurodegenerative Diseases and Geriatric Psychiatry, University Hospital Bonn, 53127 Bonn, Germany; michael.wagner@uni-bonn.de (M.W.); kathrin.heser@ukbonn.de (K.H.); 3 German Center for Neurodegenerative Diseases (DZNE), 53127 Bonn, Germany; 4 Department of Primary Medical Care, University Medical Center Hamburg-Eppendorf, 20251 Hamburg, Germany; h.koenig@uke.uni-hamburg.de (H.-H.K.); a.hajek@uke.de (A.H.); 5 Working Group Medical Statistics and IT-Infrastructure, Institute for General Practice, Hannover Medical School, 30625 Hannover, Germany; wiese.birgitt@mh-hannover.de

**Keywords:** healthy ageing, gender-specific perspectives, gender-specific interventions

## Abstract

(1) The rising proportion of older adults in the population represents a challenge for the healthcare system. Women and men age differently. This study aims to examine gender-specific characteristics of health in old age from male and female perspectives. (2) Two focus groups were formed in this qualitative study of older (70+) women (*n* = 10) and men (*n* = 8) in accordance with the theoretical framework of the World Health Organization (WHO) on healthy ageing determinants. The data were audio recorded and fully transcribed. Qualitative content analysis was performed using MAXQDA. (3) In both focus groups (average age: women 77.1 years, men 74.9 years), gender-specific characteristics regarding healthy ageing were discussed. Women focused on healthy eating, while men focused on an active lifestyle and meaningful activities. Physical and social activities were considered as important for healthy ageing in both groups. (4) Important gender-specific characteristics of health in old age were identified and recommendations for gender-unspecific and gender-specific recommendations were derived. The results provide important information for promoting and maintaining health in old age. Women and men show both similarities and differences in terms of health-related needs and individual experiences. We suggest gender-specific features in nutrition and health programs for older adults.

## 1. Introduction

Due to an increase in the world’s demographics, the proportion of adults who are 65 years and older in populations will increase significantly in the next decades [1]. This is expected to raise costs and to pose challenges to the healthcare system [2]. According to the WHO, healthy ageing is defined as “the process of developing and maintaining the functional ability that enables well-being in older age” [3]. The heterogeneous process of ageing includes sex based differences that accumulate over the lifespan and encompass health and lifestyle factors that impact ageing [3,4,5]. Women live longer, but report greater impairment in their functional, physical, cognitive, and social abilities [6,7] as well as in their subjective well-being and physical health [8]. There is also evidence of sex differences in individual dispositions, lifestyle and health behaviors. For example, generally speaking, men are physically more active at older ages, but engage in more risky health behaviors [9,10,11].

Some gender differences in health and longevity are preventable and modifiable through interventions and can, therefore, be understood as health inequalities which are “unnecessary, avoidable, unfair, and unjust” and require remedial action [3,5]. These health inequities between women and men are caused by factors that can be improved. These modifiable determinants include differences in gender roles, health behaviors, and health care delivery [12]. Thus, there is a need for interventions that positively impact these modifiable determinants to create greater health equity.

Little is known about these principally modifiable determinants in old age, as older adults are often underrepresented in research [13]. Therefore, this study investigates age-specific key themes and gender-specific characteristics (both differences and similarities) of healthy ageing from two significant stakeholder perspectives, namely older women and men, and gender-specific recommendations are provided. This study provides answers to the following research questions:

1. What opinions and attitudes exist about healthy ageing among women and men aged 70 and older?
2. What gender-specific characteristics can be identified regarding healthy ageing in women and men aged 70 and older?
3. What gender-specific and cross-gender recommendations for the promotion of healthy ageing can be derived?

## 2. Materials and Methods

### 2.1. Design

This study uses an exploratory qualitative approach employing two homogeneous gender focus groups and is reported according to the Standards for the Presentation of Qualitative Research Results (SRQR) [14]. The semi-structured interview guide was developed in a qualitative research workshop, in which questions were derived deductively based on the WHO theoretical framework on healthy ageing developed by Sadana et al. [5]. This theoretical base combines root causes, structural determinants, intermediate determinants and outcomes, as well as health outcomes of healthy ageing.

### 2.2. Study Sample

To be included in the study, participants had to be to be at least 70 years old and be fluent in German. While “old age” is commonly defined as being 65 years and older [1], the inclusion criteria began at 70 years old because we were interested in post-retirement experiences in particular. As the typical retirement age in Germany is 67 years [15], the cut-off of 70 years allowed us to be relatively sure that retirement had already taken place. This way participants had had experience in adapting to new life circumstances and this topic could be investigated.

Participants were recruited from the community in Leipzig, Germany. via the Senior Citizens’ Advisory Council of the City of Leipzig and posters in supermarkets, sports clubs and facilities for older adults. Participants were asked to contact the study organization directly by e-mail or telephone if they were interested in participating. Potential participants were sent detailed study information, a consent form, an account data form for the transfer of the expense allowance, and a sociodemographic questionnaire, in advance, by post. Eighteen participants (10 women, 8 men) fulfilled the inclusion criteria and consented to participate. Participants were assigned to the male or female focus group based on gender self-identification.

Participating men were on average 74.9 years old (standard deviation (SD) = 2.8) and participating women on average 77.1 years (SD = 4.1) (see Table 1). Sociodemographic data (gender, age, marital and living status, educational level and employment situation) were collected using a short anonymous questionnaire that had been filled out in advance at home. To ensure confidentiality, personalized data were immediately separated from questionnaires and kept separately.

### 2.3. Implementation and Analysis

Two focus groups comprising men (*n* = 8) and women (*n* = 10) were conducted in October 2019 in a seminar room at the Institute of Social Medicine, Occupational Health and Public Health in Leipzig. Both focus groups were moderated by a trained scientific research associate who was supported by a research assistant. Each discussion was introduced with a short oral presentation on the topic of health and age.

Both focus groups followed the same general structure. Firstly, the participants were asked about their understanding of healthy ageing. Then, they were asked about basic causes of healthy ageing (biological and social conditions) and about intermediate determinants (lifestyle and health behaviors, individual actions to promote healthy ageing, personality traits, social contacts and the influence of general conditions). As one objective of the study is to derive recommendations for promoting healthy ageing, participants were also asked to discuss structural determinants of health (medical care and public health promotion services). Finally, for each topic block, participants were asked about differences between men and women regarding the respective field.

The group discussions were audio-recorded, fully transcribed and content-analyzed using MAXQDA 2018, according to a protocol for qualitative analysis as outlined by Mayring and Fenzl [17]. Two trained scientific project members with experience in qualitative research independently coded and revised both interviews. The derivation of the coding scheme followed a combined deductive and inductive approach (deductive from the model-based interview guide, inductive from the audio material). Consensus and methodological rigor were established with mutual agreement during the intermediate steps (pre-encoding, revision, recoding and coding) and in a final group discussion. After this, the categories were reviewed, with examples from the transcripts. All codes are provided in Table 2.

To derive recommendations, two research assistants with qualitative expertise first identified themes. They then matched recommendations to themes, which were specifically mentioned by the participants. Next, a qualitative research workshop was conducted with experts from the fields of psychology, psychotherapy, sociology and nutritional sciences. The expert panel reviewed the topics and the assigned interventions and reached a consensus. Additional interventions were also brought forth and national and local considerations were integrated. The following themes were identified: nutrition, sports, social contacts, preventive health care, lifelong learning, meaningful activity, cognitive skills training, life events, and structural conditions. General recommendations suitable for both genders, as well as recommendations that take gender-specific aspects into consideration, were derived according to the findings.

## 3. Results

The key results are presented below (for detailed results see Appendix A). The participants discussed the meaning of healthy ageing, lifestyle and health behaviors, individual actions to promote healthy ageing, the influence of personality traits, the role of social contacts, structural conditions, life incidents, biological factors, medical conditions, health care and public health promotion services, and public infrastructure.

### 3.1. Opinion and Attitude Differences

Women expressed a generally positive attitude towards ageing and emphasized that the perception of old age has changed significantly in the last decades:

“I have a photo of my grandmother at home. On the back of the photo it says, ‘Grandma in her 63rd year.’ I’m 71 now (…) That’s two earlier generations and it’s a totally different image. (…) She was very old at 63. And that’s what I think is nice, that the people in this group right here, we are all different”.

(female participant)

For men, ageing was associated with disease and struggle:

“Yeah, you’re certainly aware that in old age you will always have little aches and pains, sometimes maybe something worse too”.

(male participant)

Both emphasized that being proactive and optimistic as well as to adaptive to changes due to ageing were important:

“I noticed with a friend that he became a bit frail and then began having difficulty walking. And then he stopped walking altogether and it continued in this way, and then he was dead. Yes, it happens very quickly at a certain age. And I think you should try to avoid that. Don’t give up, but fight. Fight every day”.

(male participant)

According to all participants, healthy ageing has genetic components, but one’s own actions can influence the course of ageing to a great extent.

“Of course, staying healthy is partly down to your genes. But I do think it’s important to take a lot of initiative yourself”.

(female participant)

In terms of a healthy lifestyle, women emphasized the importance of healthy eating:

“Yeah, my husband always says, ‘my wife cooks and that’s why I’m healthy.’ He doesn’t take care of such things” (female participant). (“No, men don’t take care of such things.”).

(another female participant)

In contrast, men focused on an active lifestyle and meaningful activities:

“Intellectual activity is important, as I said. But in my experience, you shouldn’t be just killing time with it, so to speak. Whatever it is you do, it should serve some kind of purpose. You should have a task, as they say”.

(male participant)

Social activities, including intergenerational contact, were considered important for healthy ageing by both women and men:

“Of course, the social environment is important. So many of us, I, in particular, sit at home alone all week without any contacts at all. There’s a nice saying: take time for your friends, otherwise time will take your friends”.

(male participant)

However, from the perspective of both genders, maintaining and cultivating social contacts was easier for women:

“Well, there are big differences. We are simply socialized differently, we women. We are more communicative, we are more open. And the men, they withdraw”.

(female participant)

Physical activities were considered important by both groups, but women and men described different motivations for exercising and doing sports. Women associated sports with social interaction, fulfilling leisure activities, and maintaining well-being:

“Yes, I am also a member in two exercise groups and we do a lot of things together. We often go on trips together or have dinner together. We play cards, things like that. We celebrate milestone birthdays”.

(female participant)

In contrast, men were motivated by generating a “sense of achievement” and preventing diseases:

“Until my heart attack about 20 years ago, I used to drive to the bakery. I didn’t get any exercise at all. I hated exercise. I still hate it, but I do it. I go to a cardio class once a week. I also go to the gym and exercise for half an hour”.

(male participant)

Women and men generally rated health care conditions as essentially positive. This is evident in the responses given by the participants:

“Our healthcare system is actually excellent, I would say. We really can’t complain about it. We live in luxury here” (female participant). “And I can’t complain at all. It’s all going well as far as medical care is concerned”.

(male participant)

All participants say they took advantage of preventive checkups. Nevertheless, both wished for more preventive services for older adults. Mammograms for women over 70 years old was specifically mentioned since this screening is not supported by health insurers. Similarly, older people are excluded from other preventive health services which promote well-being and health instead of focusing on pre-existing diseases and physical consequences of aging, as can be seen in this example:

“Yes, because everything comes to an end at 70. No more gynecological checkups, nothing. You’ve taken advantage of all the preventive services. But that’s all gone”.

(female participant)

Men emphasize the need to differentiate between genders and age groups when it comes to medical care and medication:

“It is certainly important to make a difference between medication for older and younger people (…) In many cases it has been found to be wrong. And differentiating between medication for women and men, which is also not being done”.

(male participant)

For women, caring for relatives and the death of significant others were discussed as elemental experiences of old age:

“Most often you’re caring for your spouse, or you’re having to deal with the fact that the end of your spouse’s life is not far off. That’s quite a challenge”.

(female participant)

Men considered retirement to be a particularly critical life event:

“You can read about it, it’s typical for men of retirement age. And as I said, I had a hard time coping. But thank God, with social support and also the help of my family, I’ve made it through so far”.

(male participant)

Regarding structural conditions, both women and men saw differences between urban and rural areas. Rural areas were considered to be worse for older people:

“I moved to T. (…). And I’m really glad to have relocated at an older age. Let me tell you, the village life is not what it used to be. It’s so difficult to live in a village”.

(female participant)

Women stated that they benefit from contacts with neighbors but felt that some of these contacts were diminishing:

“People walk up the stairs and disappear into their flats. With some I even have the impression that should I open my door, they would quickly close theirs. That has changed a lot”.

(female participant)

Men focused more on insufficient transportation:

“And yes, well, I still like driving. But I would also be happy if public transportation were good enough to allow me to leave the car at home more often”.

(male participant)

Both women and men also considered the pursuit of hobbies and adult education to be an important prerequisite for healthy ageing, as can be seen in this example:

“But I play poker seriously. The first few years after my retirement, I took courses at the adult education center. A computer course and an English course and such”.

(female participant)

Women emphasized that full participation in public life depends on sufficient financial resources:

“That’s what I was just about to say. You need some money if you want to keep busy three days a week”.

(female participant)

Men expressed the wish for specific offers for male older adults, as existing offers were often perceived to be aimed to a female target group:

“But what I’m trying to say is that something probably needs to be done because, on average, we men are being forgotten. There are a lot more opportunities and things for women. There’s this and there’s that. Men have other interests, generally speaking”.

(male participant)

### 3.2. Recommendations

A guiding principle of recommendations should be to use the potential of older people by integrating their experience and resources (e.g., their skills earned in their professional life and through their hobbies, or their free time). Offers for older adults should ideally emphasize reciprocity by providing win–win situations for children, adolescents, younger and older adults (e.g., homework help, repair cafés, coaching a sport, or other intergenerational offers). Many recommendations (e.g., soccer coaching for children by a retired coach or involvement in neighborhood gardens) have the potential to address many purposes at once (e.g., performing a meaningful task, strengthening social integration, promoting one’s own physical activity). Offers gain acceptance and effectiveness the more specific they are to regional characteristics (e.g., senior afternoons with local crafts or cooking evenings with local and seasonal specialties). Further considerations include: the degree of urbanization vs. rurality (e.g., what kind of cultural preferences are common in the place? What are the transportation options? Do older adults need pick-up and shuttle services?) and gender specifics (e.g., offer not only handicraft arts, but also craftsmanship afternoons; target men and encourage their social activity; offer workshops on cooking specifically for men who often have not learned to cook). For an overview of the recommendations, see Table 3.

## 4. Discussion

In both focus groups, participants were heterogeneous in their age distribution and therefore part of different age cohorts. The youngest had not had personal childhood experiences during World War II, while the oldest in the group had experienced years of childhood and adolescence socialization during the National Socialist dictatorship. Nevertheless, all study participants had experienced incisive social upheavals including various forms of government, comprehensive changes in gender roles, significant medical progress and modifications in the concept of old age. This historical context, in conjunction with the broad age distribution, could presumably inform very different attitudes toward healthy aging [18]. These cohort differences could not be explored further in the context and analysis of the group discussion but should be kept in mind when drawing conclusions.

The aim of this qualitative study was to identify gender-specific characteristics of healthy ageing and to derive recommendations to promote positive health attitudes and behaviors. The topics and needs mentioned are consistent with most of the research literature on healthy ageing (e.g., [19]). However, in contrast to previous research by Bryant et al. [20], female participants in our study expressed a more positive image of the ageing process compared to men, who primarily associated ageing with negative consequences. This should be addressed in gender-specific offers (e.g., gender specific nutrition courses for men who want to learn to cook healthily after the death of a spouse; or projects such as neighborhood gardens and repair cafés, which could be more attractive to older men than the existing offers that can sometimes be perceived as more appealing to women). A positive image of ageing is an important psychological resource in assessing life satisfaction. Further, self-perceptions of ageing influence the success of health-promoting programs [21,22,23,24]. Therefore, psychoeducational programs on age-related biological, psychological, and social change can positively influence the image of ageing and potentially improve functional health [25,26].

Although all participants agreed that biological factors had an influence on ageing, women and men agreed that nutrition (e.g., eating fruits and vegetables, cooking at home, limiting alcohol), exercise (e.g., rehabilitation sports, walking), and mental activities (e.g., reading, sudoku) play an important role. Further, older adults actively contribute to healthy ageing and reduce the negative effects of aging by eating healthfully, maintaining an active lifestyle and keeping mentally fit, as is consistent with the findings from Harmell et al. [19]. Our study participants emphasized the need to adapt to changes in health and to remain active. This attitude of actively taking part in a healthy ageing process sets the stage for successful programs. Initiatives to promote healthy ageing should integrate gender-specific aspects and generate social contacts at the same time. Both women and men regarded maintaining and generating social contacts as important for quality of life in old age, but assumed that this would be easier for women, which is in line with existing research [8]. In addition, contact with younger generations was seen as a supportive resource in ageing, in accordance with Teater [27]. Gender-specific aspects should be included; special offers for men should be created. Recommendations to promote social contacts for older adults could, likewise, be based on cultural, intellectual, athletic and voluntary activities. The positive impact of meaningful activities was particularly emphasized by the men in our study, which is consistent with findings from Hajek et al. [28], who emphasize the positive effect of volunteering on well-being.

Study participants reported different motivations for exercising: older men focused on improving their performance and preventing diseases, while older women wanted to maintain mental and physical fitness for their own well-being (to do “something for themselves”). Empirical research on gender-specific motivation for physical activity is still deficient and includes open questions (e.g., [29,30]); however, for both genders, social interaction as well as enjoyment of the activity appear to be greater motivators than performance goals, when compared with younger age groups [31,32]. Programs to promote physical activity should address both motivations, offer a pleasant atmosphere, create individual health benefits, and provide opportunities for social exchange [29,33].

Although women and men were very satisfied with health care, they wanted more preventive services, which is contrary to the state of research with regards to men [34,35]. Further, men wanted gender-specific aspects to be considered by physicians when prescribing a medication and determining its dosage. Thus, drug approvals and their associated studies should consider gender-specific differences (e.g., are some medications more effective in a male body, or do men need a higher dosage than women).

Targeted offers could support older adults in coping with critical life incidents. For women, illnesses, caregiving and the death of relatives is particularly impactful, in line with the research of Stein et al. [36]. For men, retirement plays an important role.

There was agreement between both women and men that environmental conditions are important in ageing. Although all participants lived in an urban area, they reported that in rural areas older people experienced limitations in mobility, health care, and cultural, sports and social opportunities, thereby hindering healthy ageing. Unfavorable environmental conditions also prevent older adults from engaging in physical activity [29,33,37]. When designing programs, accessible and barrier-free locations and age-appropriate times of day should be taken into account [38]. Consistent with Paz et al. [39], interviewees emphasized that participation in activities requires sufficient financial resources. Thus, offers should cost as little as possible or be free of charge [33,37].

A strength of this study is the focus on women and men 70+. Older people are underrepresented in empirical research due to recruitment and participation problems [40,41]. There are also some limitations due to sample size and selection bias. Participants who agreed to participate in the study may have had a high interest in the topic of healthy ageing. Therefore, sample selection effects cannot be completely ruled out. Since socio-economic status was not assessed, it was not possible to obtain an overview of the participants’ financial situation. Assessments and recommendations may, therefore, be limited. Although some of the participants have had previous life experiences in a rural area, as well as acquaintances and relatives there, they all now live in an urban area. The perspective of rural seniors has, therefore, not been sufficiently taken into account. Also, by requiring sufficient German language skills to participate in the discussion, migrant and minority ethnic groups were not represented and generalizability is also limited in this regard.

## 5. Conclusions

The current study provides important implications for practice and care offers. As the perception of ageing has improved over recent years, especially for women, these gains should be extended to men. Ageing could be embraced also by men, as a phase of life full of opportunities. In developing programs, the need for intellectually stimulating and meaningful activities and for (intergenerational) social contact should be addressed and possible side-effects emphasized (e.g., the possibility of building up social contacts while engaging in physical activities). Gender-specific needs and age subgroups in the elderly population also need to be considered in medical care.

Significant research implications also emerge. Recommendations developed in this study were guided by the principle that they should be based on the needs of the community. These recommendations should be further developed and analyzed concerning their acceptability, feasibility, and effectiveness. It would also be interesting to examine whether the same views, needs, and requirements for recommendations would emerge from a rural sample or among adults 70+ from migrant or other minority groups (with group discussions in other languages). Since men often seem to perceive existing services and programs for older adults as women-specific, research should explore what offers men would like to see and how existing offers can be improved so that they also feel addressed. As the focus group discussions took place in October 2019, prior to the COVID-19 pandemic, it would be of great interest to investigate possible changes in attitudes toward healthy aging and implications for recommendations in a follow-up study.

## Figures and Tables

**Table 1:** Sociodemographic characteristics of the focus group members.

	Focus Group: Women		Focus Group: Men	
*N*	10		8	
Age (Mean, (*SD*))	77.1	(4.1)	74.9	(2.8)
Education (*n*, (%)) ^a^				
Low	2	(20.0)	0	(0.0)
Middle	4	(40.0)	1	(12.5)
High	4	(40.0)	7	(87.5)
Living situation (*n*, (%))				
Alone	7	(70.0)	2	(25.0)
With partner	3	(30.0)	6	(75.0)
Marital status (*n*, (%))				
Married	2	(20.0)	6	(75.0)
Single	0	(0.0)	0	(0.0)
Divorced	3	(30.0)	1	(12.5)
Widowed	5	(50.0)	1	(12.5)

Note. ^a^ According to Casmin-Classification [16].

**Table 2:** Analytical codes and key quotes.

MC1: Meaning of ageing	SC1: Healthy ageing	W: “Yes, I would say that life as you know it continues without diminishing, I would say.” M: “So the most important thing is just that you are physically fit, that you are mentally moderately fit and fit for quite a long time and then die—fast as lightning.”
	SC2: Ageing process	W: “I have a photo of my grandmother at home. On the back of the photo it says, ‘Grandma in her 63rd year.’ I’m 71 now (…) That’s two earlier generations and it’s a totally different image. (…) She was very old at 63. And that’s what I think is nice, that the people in this group right here, we are all different.” M: “Yeah, you’re certainly aware that in old age you will always have little aches and pains, sometimes maybe something worse too.”
MC2: Lifestyle and health behaviors	SC1: Hobbies	M: “In my opinion, you should somehow get a sense of accomplishment (…) That you can say, well, I did something useful, I didn’t just kill time.”
	SC2: Social activities	W: “You also get to know a lot of people there, but the most important thing for me is actually the friends I’ve had from a young age. And there, we are such a great community, who also/who not only celebrate together, but also help each other.” M: “So I just wanted to say that if you are lucky enough to have such contacts now with younger people, that is important. Not just ready to withdraw, as was said earlier (…). No, you have to get out into the world, of course.”
	SC3: Nutrition	W: “I eat but, for example, I know that pork is not good for me. Of course, I don’t eat it anymore. But overall, I try not to eat fatty foods because our metabolism slows down a little bit now.” M: “I always say, you try to live in such a way that you can’t blame yourself, that you can say you live reasonably healthy. Everything in moderation. That’s/whether that’s alcohol, whether that’s exercise, whether that’s food.”
	SC4: Risk behavior	W: “But for example, I have high blood pressure, but I’m not going to be dissuaded from drinking my little wine, right? I’ll decide that myself.” M: “I mean, what‘s harmful? Beer is healthy. Too much beer is unhealthy. Hard liquor is healthy. Too much hard liquor is unhealthy. Smoking, well, I never did.”
	SC5: Healthy lifestyle	W: “How do I stay fit and how can I not become a burden to other people? I mean, other people help but I can’t give up on myself.” M: “I noticed with a friend that he became a bit frail and then began having difficulty walking. And then he stopped walking altogether and it continued in this way, and then he was dead. Yes, it happens very quickly at a certain age. And I think you should try to avoid that. Don’t give up, but fight. Fight every day.”
	SC6: Differences women/men	W1: “Yeah, my husband always says, ‘my wife cooks and that’s why I’m healthy.’ He doesn’t take care of such things.” (W2: “No, men don’t take care of such things.”). M: “As I said, there are sports that are not really sports in the strict sense, that are primarily played by men.”
MC3: Own actions to promote healthy ageing	SC1: Social contacts	W: “Hiking, for example. So when I retired, I started a hiking group with female friends.” M: “Of course, the social environment is important. So many of us, I, in particular, sit at home alone all week without any contacts at all. There’s a nice saying: take time for your friends, otherwise time will take your friends.”
	SC2: Bodily fitness	W: “Yes, I live near this wonderful swimming center. So, that’s where I go. I can tell from the 10-times discount cards that I was there 62 times this past summer.” M: “Until my heart attack about 20 years ago, I used to drive to the bakery. I didn’t get any exercise at all. I hated exercise. I still hate it, but I do it. I go to a cardio class once a week. I also go to the gym and exercise for half an hour.”
	SC3: Actions to stay mentally fit	W: “But I play poker seriously. The first few years after my retirement, I took courses at the adult education center. A computer course and an English course and such.” M: “Intellectual activity is important, as I said. But in my experience, you shouldn’t be just killing time with it, so to speak. Whatever it is you do, it should serve some kind of purpose. You should have a task, as they say.”
	SC4: Actions for emotional well-being	W: “Yes, I am also a member in two exercise groups and we do a lot of things together. We often go on trips together or have dinner together. We play cards, things like that. We celebrate milestone birthdays.” M: “As I said, you have to create a hobby.”
	SC5: Motivation	W: “Yes, well, you also have a certain personal responsibility for your life. And my aim has always been, I don’t want to be a burden on my daughter and grandchildren one day.” M: “Don’t stop, but fight. Fight every day.”
	SC6: Differences women/men	W: “Well, there are big differences. We are simply socialized differently, we women. We are more communicative, we are more open. And the men, they withdraw.” M: “There are fewer sports groups for men. Besides, as I said, women have a longer life expectancy anyway. (…) It’s difficult to convince someone to do something that they don’t want to do.”
MC4: Influence of personality traits on healthy ageing	SC1: Personality traits	W: “Well, thank God, the good Lord has given me a sunny disposition. And I also have many aches and pains, but I bear them with a bit of humor.” M: “I’m more of a pessimist in some ways. But maybe that’s also due to Parkinson’s now. But it’s dragging me down. Optimism and attitude are important.”
	SC2: Differences women/men	W: “If I look at my husband, he’s one to/his glass is three-quarters empty.” M: “Women are more outgoing about that than men. In general. Women say, ‘Yeah, I don’t got it yet. Can you explain me that again?’ And a man says, ‘I can’t.’ It’s stupid.”
MC5: Role of social contacts	SC1: Social network	W: “That’s also where I go to exercise. I go to Pilates three times a week. You can get to know a lot of people there, too, but the most important thing for me is that I see friends that I’ve had since I was a young person.” M: “So, the other problem, you have a group of people that started at the same time, and then bringing in new ones, that’s not so easy.”
	SC2: Social support	W: “This is work. That’s exactly how it is. Like my friend, for example, whose husband is dying. I cook for her, then I drive her to the clinic. Today I am going shopping with her afterwards. That’s what friendship is all about. That’s what I mean by friendship.” M: “But thank God, with social support and also the help of my family, so far I have overcome everything.”
	SC3: Differences women/men	W1: “Yes, you can see it, too, women traveling together. Women going out for walks together. But it’s almost impossible to see two men together.” (W2: “Village pub.”) “They no longer exist, where they used to sit together and chat. They sit at home and/” M: “And they (transcriptor’s note: women) look for friends then somehow. They were just bump into them. Men are (transcriptor’s note: incomprehensible), that they tell their life to a complete stranger (transcriptor’s note: incomprehensible). Men are different. But the women, they do that and/”.
MC6: Social framework conditions	SC1: Finance	W: “That’s what I was just about to say. You need some money if you want to keep busy three days a week.” M: “Are people doing something here (transcriptor’s note: volunteering) out of pure interest or are they doing it for financial reasons? And that’s where I think the commitment declines a lot.”
	SC2: Neighborhood (social integration)	W: “People walk up the stairs and disappear into their flats. With some I even have the impression that should I open my door, they would quickly close theirs. That has changed a lot.”
	SC3: Urban/Rural	W: “I moved to T. (…). And I’m really glad to have relocated at an older age. Let me tell you, the village life is not what it used to be. It’s so difficult to live in a village.”
		M: “Out in the countryside, the most important thing is a car. Without a car, you’re completely stuck out there.”
	SC4: Mobility	W: “And I then go by S-Bahn or by tram, because parking in the city center makes no sense.” M: “And yes, well, I still like driving. But I would also be happy if public transportation were good enough to allow me to leave the car at home more often.”
MC7: Life incidents		W: “Most often you’re caring for your spouse, or you’re having to deal with the fact that the end of your spouse’s life is not far off. That’s quite a challenge.” M: “You can read about it, it’s typical for men of retirement age. And as I said, I had a hard time coping. But thank God, with social support and also the help of my family, I’ve made it through so far.”
MC8: Biological factors		W: “Of course, staying healthy is partly down to your genes. But I do think it’s important to take a lot of initiative yourself.” M: “Women age better than men. I wonder why they age. Genetically. Genetic causes are primarily responsible.”
MC9: Medical history		W: “I had a lot of trouble at first, that one time. I don’t want to talk about that. So, I managed that situation with the help of my children—everything is resolved; I feel much more comfortable. Then I sorted out my circle of friends.” M: “If I forget to take my medication for half an hour—so I have to take it every four hours—I have a feeling of inner restlessness that I have to compensate for by movement. So I can’t rest either. (…). And that’s where I have my so-called activity.”
MC10: Health care and public health promotion services	SC1: Utilization	W: “Yes, I’ll get vaccinated (transcriptor’s note: flu vaccination). It won’t hurt.” M: “Yes. I think if you take advantage of the preventive care services, there’s nothing wrong with that, I think. But there are a lot of men who don’t go to the doctor at all, for example.”
	SC2: Desired support services	W: “But, what I would prefer would be to not go to the doctor for years, then to get a prescription for a short stay at a health resort.” M: “And when you say rehabilitation. I should have gone to an inpatient rehabilitation. And that was refused by my health insurance. You have to appeal the decision on your own.”
	SC3: Need for improvement	W: “Yes, because everything comes to an end at 70. No more gynecological checkups, nothing. You’ve taken advantage of all the preventive services. But that’s all gone.” M: “It is certainly important to make a difference between medication for older and younger people (…) In many cases it has been found to be wrong. And differentiating between medication for women and men, which is also not being done.”
	SC4: Access to treatment services	W: “But long waiting times for appointments with medical specialists is also a problem.” M: “And I’ve had two nose surgeries. After the first one, I immediately went to a rehabilitation center. And now in 2017, after the second one, I had to change otolaryngologists. And he said, ‘You’ve been my patient for such a short time. You won’t be able to go to a rehab center. So that was rejected. But that had been helpful for me the first time, rehab treatment. This time, I had no chance at all.”
	SC5: Quality of health care	W: “Our healthcare system is actually excellent, I would say. We really can’t complain about it. We live in luxury here.” M: “And I can’t complain at all. It’s all going well as far as medical care is concerned.”
	SC6: Differences women/men	W: “Yes, the men have to be pushed. The men have troubles keeping up.” (Transcriptor’s note: with preventive examinations) M: “But, after all, there are many men who don’t go to the doctor at all, for example.”
MC11: Use of public services and infrastructure	SC1: Education	W: “But I play poker seriously. The first few years after my retirement, I took courses at the adult education center. A computer course and an English course and such.” M: “Yes, and there are also offers in the community centers for senior citizens, yes, you can go there. And, yes, for us old people, I say, there we were taught how to use the device (transcriptor’s note: smartphone). You shouldn’t refuse, because it is really important and really okay. But I have to want it myself. If I don’t want/”
	SC2: Cultural offers	W: “So, for example, the city library organizes great free events. But in the evenings. Or ‘Haus des Buches’ (transcriptor’s note: local venue that offers readings). ‘Haus des Buches’ at ‘Gerichtsweg’ is also great. And that, yes, I think that also helps a bit to keep up.” M: “Or you go to the ‘Gewandhaus’ (transcriptor’s note: a local concert venue for classical music) once a month, even if that sometimes hurts a bit. But you can always have a beer afterwards if necessary. So art and cultural offerings. Men are certainly a bit behind in that respect. So according to my experience. They have a hard time with that.”
	SC3: Sports	W1: “I always went to rehabilitation sports, I must say. And there it was nowhere near as full as there.” (W1: “Well, but you go to the group?”) “But yes.” (W1: “Once only?”) No, I go to three groups.” (W1: “That’s nice.”) “And so, three times, of that I do twice in a row and another day the third group, that I don’t have to go there three times a week. That would be too much. But going twice is fine.” M: “Regarding dancing in general, it’s usually the women who are best. In square dancing, it’s the men. Why is that? I was quite surprised when I took a closer look at this phenomenon, that in the beginning women predominate. And the better my own dancing became, the fewer women there were. Why? Square dancing is mathematics.”
	SC4: Differences women/men	M: “But what I’m trying to say is that something probably needs to be done because, on average, we men are being forgotten. There are a lot more opportunities and things for women. There’s this and there’s that. Men have other interests, generally speaking.”

Notes. MC = main categories; SC = subcategories; W = woman; M = man.

**Table 3:** Recommendations to promote healthy ageing from the perspective of women and men.

Topic	Examples and Suggestions as Mentioned in Focus Groups		Recommendations Derived in a Qualitative Research Workshop
	Women	Men	
Nutrition	Promotion of healthy diet	Promotion of healthy diet	Offer nutritional counseling targeting older adults (e.g., nutrition courses for men at adult education centers; cooking together in assisted living and nursing homes) Provide information on healthy nutrition in old age via print and digital media directed toward older adults (e.g., “recipe of the day” by mail or app with shopping list, tailored to the eating habits and dietary requirements of older people) Consideration of gender-specific characteristics: making offers that appeal specifically to men
Sports and exercise	Sports Fitness center Rehabilitation sports Hiking Gymnastics Swimming Yoga Walking Gardening Walking the dog	Sports Fitness center Rehabilitation sports Hiking Cycling Gardening	Create of specific exercise and sporting offers for older adults (e.g., in adult education centers, local senior and sports groups; creating opportunities to train kids and adolescents; neighborhood garden projects; neighborhood dog walking) Provide information about existing services in the region via print and digital media directed toward older adults (e.g., in daily newspapers, city magazines and local newspapers, with posters in supermarkets and flyers in mailbox, and via local radio and television) Consideration of gender-specific characteristics: since there are different motivations for participating in sports programs (women: “do something for myself” vs. men “sense of achievement”), this should be taken into account when creating and advertising sports programs
Social contacts/Social activities	Active social lifestyle Cultivate social contacts Social activities: e.g., card games, conversation course, literature circle	Active social lifestyle Hobbies Social activities: e.g., chess, bowling, dancing	Expand and promote services for older adults (e.g., advisory councils in cities, cafés in neighborhoods for older adults or “senior citizens breakfast/coffee round table”, promotion of visitor services for people who are limited in their mobility due to physical impairments; joint knitting or carving afternoons) Create opportunities that take into account specific urban and rural conditions (e.g., pick-up service for trips to cultural or sporting events in case of poor local transport or longer distances; local history clubs; participatory research projects on local history; neighborhood festivals with activities for older adults; practicing local crafts or knitting together; museum or city tours from/for older adults) Improve on-line access and technology training (e.g., use of online forums for older adults; promotion of digital skills like “smartphone courses”; installation of internet access facilities in assisted living or retirement homes) Consideration of gender-specific characteristics: create offers that make it easier for men to use them (e.g., by directly addressing men, encouraging them to participate, topics aimed at men); specifically address men, as they often do not express their feelings (common for generation at that time).
Preventive health care	Regular preventive medical checkups Preventive health cures Positive health behavior: e.g., sauna	Regular preventive medical checkups Preventive health cures Gender-specific prescribing and dosing of medications	Enhance medical care for older adults (e.g., encouraging primary care physicians to spend more time with older patients, as they are their key contact person for medical care; geriatric care centers; consulting services for the adjustment of medication; information about healthy everyday management aimed at the needs of older adults) Provide information about existing services in the region via print and digital media aimed directly at older adults (e.g., in daily newspapers, city magazines and local newspapers, flyer in mailbox) Consideration of gender-specific characteristics: information on gender-specific characteristics in the prescription and dosage of medications by general practitioners and specialists; studies on medication approvals with a stronger focus on gender differences.
Lifelong learning	Adult/continuing education	Adult/continuing education Special offers for men	Strengthen and promote adult education with course offers aimed at older adults (e.g., in adult education centers, with senior studies and public lectures) Consideration of gender-specific characteristics: e.g., specific courses for men and women
Meaningful activity	Meaningful activity	Meaningful activity Volunteer work	Create opportunities for social engagement (e.g., cross-generational offers: older adults offer help with homework or use of existing expertise of older adults while offering help in repair cafés or courses in youth centers like old printing/typesetting techniques, coaching children and adolescent sports groups; helping at the animal shelter with feeding or walking the dog; offering visitor services in hospitals themselves if physically fit; volunteer)
Cognitive skills training	Reading (public library) Crosswords Sudoku	Hobbies	Offer opportunities cognitive fitness training for older adults (e.g., reading circles and writing workshops in libraries, chess groups, memory training, quiz afternoon in a neighborhood café)
Life events	Care for relatives Death of friends and relatives	Transition from work life to retirement	Promote group activities that build social networks and strengthen social integration after a critical life event (e.g., self-help groups in general and for people suffering from loneliness; regulars’ table; bereavement café) Consideration of gender-specific characteristics: e.g., development of targeted services that address better ways of dealing with caregiving and death of relatives (women) and the transition to retirement (men)
Societal/structural conditions	Neighborhood relations Cultural offers (urban-rural gap, insufficient financial resources for the utilization of cultural offers)	Cultural offers (urban-rural gap) Mobility: use of public transport	Strengthen cultural offerings in rural areas (e.g., traveling theater, readings in the pub, field trips and excursions; exhibitions in the city hall) Offer cultural events that are free of charge or low cost (e.g., “senior citizen discount”; “pay what you can”-admission fees; local sponsorships) Promote initiatives to encourage neighborly contact and neighborhood assistance (e.g., neighborhood groups in social networks; multigenerational houses/centers; “Schwatzbank”/“Chat bench” at public places = bench with a sign indicating you would like to have a chat with a seat neighbor) Create special opportunities to maintain mobility for older adults, especially in rural areas (e.g., “Mitnahmebank”/“modern hitchhiking” = bench with a sign indicating a person would like to be given a ride; communal or private shuttle services to institutions, shopping halls or cultural places)

## Data Availability

All data generated or analyzed during this study are available from the corresponding author on request.

## Appendix A. Detailed Results According to Main Categories (MC)

### Appendix A.1. MC1 Meaning of Ageing

Women: Despite the consensus between both groups that the process of ageing was challenging, the female participants expressed a generally positive attitude towards ageing. They emphasized that the idea of old age had changed massively within two generations and that people were still fit and agile at the age of 70 to 80. Healthy ageing was associated with healthy nutrition, exercising, and an active and social lifestyle. Social exchange and integration as well as openness to new things (lifelong learning, getting to know people) were important.

Men: Among the male participants, ageing was associated with disease, survival, and struggle. Good and healthy ageing was associated with self-determination, limited disease and a painless death. In general, ageing meant adapting to the current state of health and making the best of it. Meaningful activities that had positive effects on the activity level in old age were important.

### Appendix A.2. MC2 Lifestyle and Health Behaviors

Women: A healthy lifestyle (including a healthy diet, physical and social activities, regular medical screening and learning new content) contributed to healthy ageing. Men reported caring less about healthy eating, relying on women in this regard.

Men: The male participants emphasized that being proactive was important. Maintaining physical and mental activity, as well as hobbies and meaningful tasks, were essential for healthy, active and independent ageing. After retirement, hobbies helped to structure leisure time, providing motivation, a sense of achievement and positive social and cognitive effects. Social contacts were important for an active lifestyle, as they encouraged leaving the house and were a source of inspiration for activities. The topic of nutrition was brought up to a lesser extent. It was perceived as important to be moderate in the amount of food and alcohol consumed. Smoking was rated as harmful for health. In their opinion, women maintained a more active lifestyle and were more motivated to live healthily.

### Appendix A.3. MC3 Own Actions to Promote Healthy Ageing

Women: Healthy ageing was reported to be strongly dependent on one’s own actions. There was a desire to maintain functional abilities for as long as possible, in order not to become a burden on relatives. The female participants wanted to maintain mental and physical fitness for their own well-being, and they suspected that men were primarily motivated to keep an active lifestyle by performance goals. They repeatedly emphasized the high relevance of taking the initiative in maintaining social contacts. Exercising (e.g., hiking, swimming, rehabilitation sports, gardening or walking the dog) and intellectual activities (e.g., conversation groups and literature circles) were further seen as opportunities for social exchange. Female participants perceived major gender differences in social behavior due to socialization. In their opinion, men had more difficulties in making new friends and engaging in activities. Furthermore, they assumed that men were more reluctant to ask for help. In addition, it was observed that places where male social life used to take place were now disappearing (e.g., regulars’ tables in pubs).

Men: Physical activities (e.g., dancing, cycling, bowling and rehabilitation sports) were not primarily seen as a pleasure, but as serving a specific purpose (e.g., fighting illness) or being helpful for the achievement of a certain goal (e.g., having a sense of accomplishment). The male participants could increase their motivation for activities by setting goals for themselves, generating “feelings of success” and giving their everyday life a sense of purpose. Meaningful activities that had positive effects on the activity level in old age were seen as important. There was also consensus that a social network needed maintenance and cultivation. Mental and physical activities were seen as social benefits. Men reported having more motivational problems than women in creating an active everyday life, including physical, cultural or social activities, after retirement. Women generally had a higher activity level.

### Appendix A.4. MC4 Influence of Personality Traits on Healthy Ageing

Women: It was assumed that optimistic and open-minded people find it easier to cope with the process of ageing. In addition, these personality traits also facilitated getting to know people and building up a comprehensive social network in old age. In contrast, anxiety was reported to interfere with independent ageing by setting limits to oneself. There was consensus that older men were more often seen as pessimistic and reserved.

Men: A positive attitude towards ageing was perceived as central to healthy ageing. It also facilitated dealing with health impairments and led to a more active lifestyle. Optimism was seen as a modifiable attitude that could be increased through effort and practice. According to male participants, women were generally more open-minded and more capable of dealing with frustrating experiences.

### Appendix A.5. MC5 Role of Social Contacts

Women: Female participants differentiated between types of social contacts (e.g., old and new friendships, family, and neighborhood) to a greater extent compared to male participants. Old friendships from school days were considered important for a sense of community and support. Furthermore, women considered getting to know new people as important. Contacts with peers of the same age were important because they similarly understood age-specific problems, while being in touch with younger people was inspiring. Female participants further reported fewer contacts without any emotional benefit. In general, openness and individual initiative were considered as central for establishing and maintaining social contacts. In case of an emergency, old friends and family members were considered to be major sources of support, while more recent acquaintances were considered less reliable. According to female participants, there were significant differences between the sexes in terms of social competence: men had difficulties in accepting social support and in making new contacts.

Men: Male participants appreciated social contacts and considered these stimulating and meaningful. However, fostering social contacts was often difficult for men. Meeting people of other age groups, especially younger people, was considered important. Family members were seen as an essential source of social support in times of need. Spouses typically motivated men to engage in activities. According to male participants, ageing men were more likely to withdraw and to become lonely. In their judgement, women were able to make new social contacts more quickly after social losses.

### Appendix A.6. MC6 Social Framework Conditions

Consensus: Both focus groups equally (“men” and “women”) perceived differences for older adults when comparing urban and rural areas regarding general external conditions. Rural areas were associated with poorer conditions regarding medical care, mobility, social and cultural offers as compared to urban areas, especially in comparison to “the past”. The possibility of being mobile without car by means of public transport was emphasized as a great urban advantage compared to rural life. On the other hand, more deep-rooted social interactions were an advantage in rural areas.

Women: The female participants reported diverse experiences regarding neighborhood relations in the city. In some cases, relationships with neighbors were considered anonymous, while other women reported good neighborly relations in their residential building. Good neighbor relationships were described as having been self-initiated and important to subjective well-being. The participants mentioned that financial hardship contributes to poor conditions for healthy ageing. In addition, when financial resources were low, financial support of family members was considered to be more important than cultural attractions.

Men: For male participants, neighborhood relations did not play a central role. They emphasized mobility as highly relevant: driving a car was associated with independence, and thus giving up a driver’s license was seen as a loss of autonomy and sovereignty. Better public transport connections were also desired. They criticized that there were increasing difficulties in finding volunteers for voluntary work. Here, financial interests would increasingly impact commitment.

### Appendix A.7. MC7 Life Incidents

Women: Both caring for and the death of a close relative were described as very emotionally and physically stressful events. Furthermore, these life events reduced social contacts and support. According to the participants, men would show problems in opening up to offers of support.

Men: According to male participants, retirement represented a crisis for the individual, which could be accompanied by psychological instability (depression) particularly in men.

### Appendix A.8. MC8 Biological Factors

Consensus: Both focus groups (“men” and “women”) agreed that a genetic predisposition had a certain influence on health and the quality of ageing, e.g., having a genetic predisposition to certain diseases.

Women: Female participants emphasized that, despite a genetic predisposition, one’s own initiative to stay healthy was important.

Men: Male participants saw differences in the genetic constitution of the sexes: e.g., the life expectancy of women was higher due to genetics. In their opinion, women also had healthier lifestyles as compared to men. On the other hand, the athletic performance of men was estimated to be higher compared to that of women.

### Appendix A.9. MC9 Medical History

Women: According to female participants, being ill increased the risk of loneliness. In particular, serious and chronic illnesses were linked to reduced visits from friends. Healthy old people would prefer not to think about future illnesses or their own death.

Men: Male participants reported a negative impact on quality of life due to illness as most of them had already experienced a severe illness. In their mind, diseases had a negative impact on physical fitness and resulted in a negative view on life. In addition, dealing with or fighting illnesses was experienced as dominating daily living and limiting opportunities. Nevertheless, for several subjects, the onset of illness led to a healthier lifestyle (e.g., more exercise).

### Appendix A.10. MC10 Health Care and Public Health Promotion Services

Consensus: In both focus groups, health care was rated positively in general. Women and men both desired preventive health care.

Women: Female participants were aware of the health screening options and vaccination offers. They complained that mammography screening after the age of 70 was no longer free of charge and that the communication between specialists and general practitioners was often inadequate. Legal euthanasia was discussed as desirable option. According to participating women, men were more likely to be urged by their spouses to attend preventive checkups.

Men: According to male participants, preventive check-ups were attended regularly. It was criticized that some medical services seemed to be dependent on patient status (regular vs. new patient). Better advice regarding alternative medicine (homeopathy) as well as free health check-ups when changing medication were desired. The prescription and dosage of medications was criticized as not being differentiated according to age and gender. The high volume of patients seen by physicians and, as a consequence, difficulty in obtaining doctor’s appointments were perceived as problematic.

### Appendix A.11. MC11 Use of Public Services and Infrastructure

Women: Female participants regularly used public health services (e.g., health centers, swimming pools) and cultural offers for older adults (e.g., adult education center, senior academy of the university, new media courses) and evaluated them positively. They criticized the inefficient capacity utilization of both swimming pools and fitness centers: due to a lack of staff, facilities remained closed, although swimming was a sport for every age group.

Men: Male participants also used health sport offers, but more out of a sense of duty than as recreational pleasure. Sports offers for the male target group were rated as positive. Men would also make use of cultural offers and opportunities for education. That many sporting and cultural activities for older adults were addressed toward women was criticized, and men stated that they would prefer more “male-oriented” offerings, such as repair workshops.
